# 

**Published:** 1898-10

**Authors:** 


### PERSONALS.

S. G. Burkholder, M.D., V.S., is a new addition to the Faculty of the McKillip Veterinary College. He will instruct in the Department of Meat-inspection.

Dr. J. M. Wright, of McKillip Veterinary College, will address the Interstate Association of Sanitary Boards at Omaha, November 11th, on "Glanders and Its Suppression."

Dr. R. C. Moore, of the Kansas City Veterinary College, acted as veterinarian at the Kansas City Horse Show the latter part of September. All horses were examined prior to entry, and horses with marked unsoundness were rejected.

Dr. Edwin Hogg, of Kirkwood, Pa., has purchased an interest in the practice of Dr. Harry Walters, of Wilkesbarre, the same State.

Dr. S. W. McClure, graduate of the Veterinary Department of the University of Pennsylvania, class of '98, has located at Oxford, Pa.

Dr. Harry Walters, of Wilkesbarre, Pa., is planning an ocean voyage with the hope of obtaining a restoration of his impaired health.

Dr. Leonard Pearson, State Veterinarian of Pennsylvania, addressed the Jersey Cattle Breeders' Association at Lancaster early in September.

Dr. M. E. Conard, of West Grove, was an interested visitor at the Grangers' picnic held at Williams Grove late in August.

Dr. Solomon Bock, of Denver, has been confined to his bed for a number of weeks with an attack of locomotor ataxia, but is now convalescing.

Dr. Henry Bower, of the class of '97, University of Pennsylvania Veterinary Department, has already risen in fame through his trotting horse "Lawrence" winning the purse for the three-minute class at the races of the North Penn Driving Association of Philadelphia.

Dr. M. J. Treacy, of the Eighth Cavalry of the U. S. Army, who is on a furlough and visiting Indianapolis, gave prompt and forcible denial to the slanderous publication made by an Indiana Army Chaplain, appearing in the *Indianapolis Sentinel*, against Lieut.-Col. Rush S. Huidekoper, Chief Surgeon of the First Army Corps.

Dr. E. Bovett, of Denver, is veterinarian to the city troops of Denver, Colorado.

Dr. H. J. Detmers, of Columbus, Ohio, recently resigned his connection with the State Board of Veterinary Medical Examiners of the Buckeye State.

Dr. A. E. Cunningham, V.M.D., has opened an office for practice at 215 Huntington Street, Cleveland, Ohio.

A. N. Lushington, V.M.D., formerly of Belmear Institute, Rock Castle, Virginia, has opened an office in Lynchburg, Virginia.

Dr. E. H. Landes, of Camden, N. J., has been for some time connected with the army and stationed for duty at Tampa, Florida.

Dr. H. P. Monk has become associated as House Surgeon to the Paterson Veterinary Ho-pital and Assistant to Dr. Wm. Herbert Lowe, City Veterinarian of Paterson, N. J.

Dr. D. S. White, of the Veterinary Department of the Ohio State Univerity, was recently appointed a member of the Ohio State Board of Veterinary Medical Examiners.

Dr. W. J. Martin, of Kankakee, Illinois, has lately made extensive additions to his infirmary. Among them is a new operating-room, in which has been installed a new operating-table of the latest design.

Drs. William Sheppard, Thomas G. Sherwood, and J. E. Ryder will officiate as veterinarians to the national horse show at Madison Square Garden, New York, in November next.

Dr. J. Stewart Lacock, of Allegheny, Pa., is the promoter of the Pittsburg Walker-Gordon Laboratory for the preparation and sale of "modified milk," to be prepared on physicians' prescriptions. A high-grade milk and cream will be prepared and sold. The company will employ the highest sanitary precautions; only tuberculin-tested cows and animals free from all other diseases will be allowed in the herd.

Dr. John J. Repp, of Philadelphia, an attaché of the Pennsylvania Livestock Sanitary Board, spent a week in September in the western and southern part of Pennsylvania.

Dr. F. H. Schneider, of Ashland, Pa., was a visitor to Philadelphia the latter part of September.

Dr. Maurice O'Connell, of Holyoke, Mass., has been reappointed by Governor Wolcott one of the Board of Cattle Commissioners of the "Bay State" for a period of three years.

Prof. Stiles, of the Bureau of Animal Industry, is doing good work in Germany for our livestock producers and exporters of food-products in clearing up the unjust imputations placed upon American pork.

Dr. B. M. Underhill, of Media, Pa., after a sojourn of nearly a month in Iowa and Nebraska, has returned to his practice.

Veterinarian George P. Statter, of Sioux City, Ia., is an expert polo-pony rider and player, and exhibited some very clever ponies at the Kansas City horse show.

Dr. R. Lindsey Tritton, of Richmond, Va., was recently a Gotham visitor. His son is attending the New York College of Veterinary Surgeons, having graduated from the Bethel Military Academy.

Dr. William H. Kelly, of Albany, N. Y., tarried for a number of days in Chicago on his return trip from Omaha, and enjoyed a visit to the Chicago Veterinary Colleges and association for a time with some of the "Windy City" veterinarians. Mrs. Kelly accompanied him as far as Chicago.

Dr. W. P. Phipps, of Lionville, Pa., spent some weeks in Iowa and Nebraska, where he found evidences of increasing prosperity on all sides.

Dr. Edward M. Ranck, of the H. K. Mulford Company Laboratories, was a visitor to Greater New York in October, visiting, among other places, the laboratories of the Health Bureau at the New York College of Veterinary Surgeons.

One of Colorado's prominent veterinarians is said to be the promoter and backer of a proprietary remedy company who are placing their "Every Man His Own Horse Doctor" cases throughout the State. He may be reached through Association channels under a breach of the code of ethics.

